# Favorable Lip and Oral Cancer Mortality-to-Incidence Ratios in Countries with High Human Development Index and Expenditures on Health

**DOI:** 10.3390/ijerph18116012

**Published:** 2021-06-03

**Authors:** Wen-Wei Sung, Yong-Chen Hsu, Chen Dong, Ying-Ching Chen, Yu-Chi Chao, Chih-Jung Chen

**Affiliations:** 1 Department of Urology, Chung Shan Medical University Hospital, Taichung 40201, Taiwan; flutewayne@gmail.com; 2 School of Medicine, Chung Shan Medical University, Taichung 40201, Taiwan; s0901047@gm.csmu.edu.tw (C.D.); ma611821@gmail.com (Y.-C.C.); c928714@gmail.com (Y.-C.C.); 3 Institute of Medicine, Chung Shan Medical University, Taichung 40201, Taiwan; 4 Department of Pathology and Laboratory Medicine, Taichung Veterans General Hospital, Taichung 40201, Taiwan; shuyongjen@hotmail.com

**Keywords:** lip and oral cancer, mortality, incidence, mortality-to-incidence ratio, expenditure, human development index

## Abstract

Background: The incidence rates of lip and oral cancer have continued to increase, and prognosis is associated with a country’s socioeconomic status. The mortality-to-incidence ratio (MIR) is a reasonable indicator of disparities in cancer screening and treatment. In this study, we aimed to understand the association between economic status and cancer prognosis. Methods: Data were obtained from the Global Cancer Observatory (GLOBOCAN) and the World Health Organization (WHO). The MIRs were compared to evaluate the correlation with the human development index (HDI), the current health expenditure (CHE), and the ratio of CHE over gross domestic product (CHE/GDP) disparities via Spearman’s rank correlation coefficient. Results: The results showed that Asia had the most cases and deaths. In addition, they showed a significant association (*p* < 0.001, *p* = 0.005, and *p* < 0.001, respectively) of the crude rate (CR) of incidence with the HDI, the CHE, and the CHE/GDP. However, their associations with mortality rate (*p* = 0.303, *p* = 0.997, and *p* = 0.101) were not significant. Regarding the correlation of the MIRs, the results revealed a significant association with the HDI, the CHE, and the CHE/GDP (*p* < 0.001, *p* < 0.001, and *p* < 0.001, respectively). Conclusion: Countries with higher HDI, CHE per capita, and CHE/GDP tend to have lower MIRs, which indicates favorable clinical outcomes.

## 1. Introduction

Lip and oral cancers are among the most common cancers worldwide, and their incidence rates have continuously increased in recent years [1]. Although they vary geographically, the highest incidences have been reported in South Asia and Southeast Asia [2]. Among all anatomic subsites of lip and oral cavity cancer, the tongue is the most commonly affected worldwide. The second most commonly affected site in Asia is the buccal/labial mucosa [3]. Common risk factors for lip and oral cavity cancer include tobacco, alcohol, betel quid, Epstein-Barr virus, and human papillomavirus infection [4,5]. Oral cavity cancer with leukoplakia can be diagnosed in early stages by visual examination, biopsy, or image study. Thus, early oral screening is crucial to ensure a five-year survival rate [6,7]. However, poor and less educated people with risk factors seldom obtain screening for oral cancer, and diagnosis is usually delayed [8].

Standard treatment varies according to the stage of oral cancer, including surgical resection, radiotherapy, chemotherapy, or their combination [9]. A previous study indicated that during the past 40 years, the prognosis of oral cancer has substantially improved, which has been due to the application of adjuvant radiotherapy, targeting therapy, and adjuvant chemoradiotherapy [10,11]. Recently, immunotherapy has emerged as a potential and effective treatment [9,12]. The prognosis of oral cancer is related to several factors, such as race [13], anatomic subsites [14], and diagnosed stage. Prognosis varies with disparities in socioeconomic status [15]. People with limited income and heavier oral cancer disease burden may receive less definitive therapy and have lower overall survival rates than others [16,17]. As the early detection of oral cancer is one of the most important factors that affect overall survival and prognosis [18], an affordable oral cancer examination could help early diagnosis, improve prognosis, and increase survival rates [19].

The mortality-to-incidence ratio (MIR) is calculated by crude mortality rates over crude incidence rates, which determines whether a country has a higher or lower mortality rate [20] and reveals the overall mortality rate after diagnosis of the disease [21]. Sunkara and Hébert’s study [22] suggested that the MIR could be a useful indicator for identifying regional disparities in cancer screening and treatment.

As the MIR indicates mortality after accounting for incidence, it can help in assessing the prognosis of cancer and its burden on healthcare. Thus, in this study, we evaluate health expenditures, the human development index (HDI), and the MIRs of selected countries to understand the association between economic status and cancer prognosis.

## 2. Materials and Methods

Epidemiological data on lip and oral cavity cancer, ICD-10 C00-06, were obtained from the Global Cancer Observatory (GLOBOCAN) database (https://gco.iarc.fr/today/, accessed date: 11 October 2020), which is a public access database that provides contemporary estimates of cancer epidemiology in 185 countries for 2018. The HDI was obtained from the United Nations Development Program, Human Development Report Office (http://hdr.undp.org/en, accessed date: 11 October 2020). Health expenditure data, including the per capita current health expenditure (CHE) and the ratio of CHE to gross domestic product (CHE/GDP), were obtained from the World Health Statistics database (https://www.who.int/gho/publications/world_health_statistics/en/, accessed date: 11 October 2020).

The MIR is defined as the ratio of the crude rate (CR) of mortality to the CR of incidence, as previously described [22,23,24,25]. The exclusion criteria for country selection were based on missing data in the World Health Organization (WHO) statistics (N = 12), missing data of the HDI (N = 2), and the data quality report by the GLOBOCAN (N = 110) [26]. A total of 61 countries were included in the analysis. The associations between the MIR and the HDI, the CHE, and the CHE/GDP in various countries were estimated using Spearman’s rank correlation coefficient, which was calculated using SPSS statistical software version 15.0 (SPSS, Inc., Chicago, IL, USA). Values of *p* < 0.05 were considered statistically significant. Scatterplots were generated using SigmaPlot.

## 3. Results

### 3.1. Epidemiology of Lip and Oral Cancer According to the Regions

We surveyed 339,913 new cases and 168,169 deaths from lip and oral cavity cancer in this study. The selected countries are grouped into six regions based on the continent in which they were located. The incidence and mortality case numbers, CR, age standardized rate (ASR), and MIR are presented in Table 1. The findings showed the incidence numbers in Africa (13,324), Asia (220,810), Europe (57,737), Latin America and the Caribbean (18,525), North America (25,354), and Oceania (4163). The findings also showed the number of deaths in Africa (9066), Asia (124,900), Europe (21,834), Latin America and the Caribbean (7050), North America (4424), and Oceania (895). Among all the selected regions, Asia was shown to have the highest numbers of cases and deaths, while Oceania had the lowest numbers of cases and deaths.

### 3.2. Epidemiology and Parameters of the Development and Health Expenditure of Lip and Oral Cancer in the Selected Countries

The HDI, CHE, cancer incidence, cancer mortality, and MIR of selected countries are shown in Table 2. Egypt had the lowest HDI (0.700), and Norway had the highest HDI (0.954). The CHE/GDP ranges from 3.1% (Qatar) to 16.8% (USA). The incidence crude rates in all the examined countries ranged from 1.0 in Qatar and Bahrain to 12.7 in Latvia. The age standardized incidence rate (ASR) ranged from 1.1 in Chile to 6.9 in Australia. Egypt had the lowest mortality CR (0.4), and Latvia had the highest mortality CR (6.0). Chile, Egypt, Israel, Malta, and Jamaica had the lowest age standardized mortality rate (ASMR, 0.4), and Latvia had the highest ASMR (3.2). The MIR ranged from 0.14 in Australia to 0.64 in Bahrain.

### 3.3. Association between MIR and Parameters of the Development and Health Expenditures in the Selected Countries

**Figure 1:**
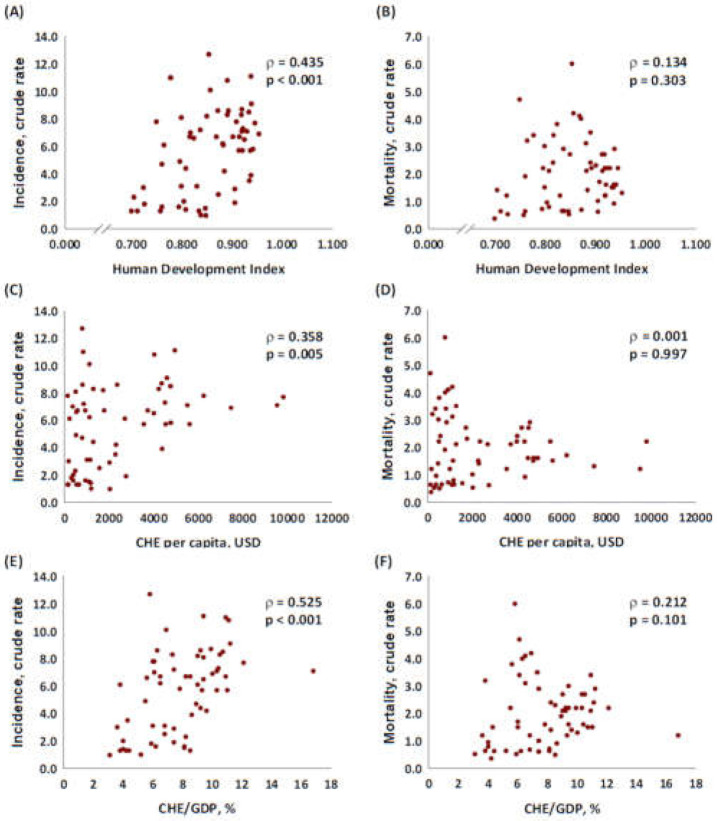
Association between HDI, CHE, and CRs of (**A**,**C**,**E**) incidence, and (**B**,**D**,**F**) mortality in lip and oral cancer.

**Figure 2:**
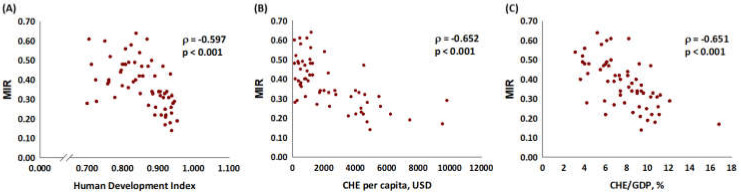
(**A**) HDI, (**B**) CHE per capita, and (**C**) CHE/GDP are significantly associated with the MIR for lip and oral cancer.

We further examined the association of incidence and mortality CR with the HDI, the CHE, and the CHE/GDP. The CR of incidence had a significant correlation with the HDI (*p* < 0.001, Figure 1A), the CHE (*p* = 0.005, Figure 1C), and CHE/GDP (*p* < 0.001, Figure 1E). The CR of mortality did not have a significant association with the HDI (*p* = 0.303, Figure 1B), the CHE (*p* = 0.997, Figure 1D), and the CHE/GDP (*p* = 0.101, Figure 1F). In addition, the results revealed a significant association of the MIRs with the HDI, the CHE per capita in USD, and the CHE/GDP with the MIR of lip and oral cancer (coefficient correlation = −0.597, *p* < 0.001; coefficient correlation = −0.652, *p* < 0.001; coefficient correlation = −0.651, *p* < 0.001, respectively, Figure 2).

## 4. Discussion

In the present study, we analyzed the incidence and mortality of selected countries. The findings showed that Asia had the most cases and deaths among all countries. These results showed the correlation of the geographic distribution of betel chewing with high oral cancer incidence and mortality. In Asia, especially Southeast Asia, chewing betel quid that has various ingredients is a prevalent oral habit [5]. Betel quid has been considered a carcinogen by the International Agency for Research on Cancer (IARC) for several years [27]. Previous results have shown that betel quid chewing has a significant negative effect on oral cancer and precancer [28]. Longer duration and higher frequency of betel quid chewing have been found to increase the risk of oral cancer [29].

Our findings showed that countries with higher HDIs and CHEs had higher incidence rates (Figure 1). This result supports the findings of previous studies that indicated the cancer incidence burden was greater in countries with higher HDIs [30]. This finding might have been because more developed countries have more comprehensive medical facilities, and patient education.

Some novel methods, such as Toluidine blue staining, brush biopsy, chemiluminescence, and tissue fluorescence spectroscopy screening, aid in the early diagnosis of oral cancer [7]. The early detection of oral cancer and public awareness of oral cancer screening are some of the most efficient ways to reduce mortality rates and contribute to better outcomes [31]. Previous results also showed that socioeconomic disparities influence oral cancer screening [8], which affects the severity at diagnosis and prognosis [32].

However, in comparing HDI, CHE, and CHE/GDP with MIR, we discovered that more developed countries had lower MIRs (Figure 2) with significant correlations. A previous study showed that MIR was a valid proxy for five-year relative survival [33], which supported that more developed countries have longer survival rates. A previous retrospective study showed that in recent decades, improved operative techniques, advanced preoperative image assessment, and efficient radiotherapy enabled a substantial increase in oral cavity squamous cell carcinoma survival rates [34]. In addition, new options, such as immunotherapy and target therapy, have become increasingly popular in treating oral cancer in advanced stages [9]. Due to the deepened understanding of the oncogenic and epigenetic pathways of oral cancer, more biomarkers for identifying invasive and metastatic potential tumors have been discovered. Moreover, based on genetic findings, there have been advances in precision therapy [35]. Thus, previous findings indicated that countries with higher HDIs and CHEs have higher early detection rates and more effective treatment, which leads to higher incidence rates and lower MIRs, which is compatible with our results.

A previous study showed that because the MIR lacked comprehensive follow-ups, it could not replace cancer survival rates or prognostic data on long-term follow-ups or cohort studies [36]. However, other studies have shown that the MIR is still a simple and efficient method for identifying cancer control [33]. The MIR has also been shown to be a suitable method for examining cancer screening and treatment programs worldwide [22].

The present study has the following limitations. First, some important risk factors for lip and oral cancer, such as alcohol consumption, smoking, betel chewing, and other oral behaviors among different countries, were not recorded or analyzed. These risk factors may play important roles in explaining and determining incidence and mortality rates among countries and regions. Second, the feasibility of using WHO rankings and CHE/GDP to represent healthcare disparities among countries was not confirmed to apply to different cancers. The data reported by GLOBOCAN were based on estimates in some countries. National healthcare systems, disparities in the access to cancer care, and insurance coverage were not analyzed. Despite these limitations, the findings showed that the MIR appears to provide more accessible data compared with long-term follow-up survival surveys.

## 5. Conclusions

The MIR for lip and oral cancer is significantly associated with the HDI, the CHE per capita, and the CHE/GDP. Countries with higher HDIs, higher CHE per capita, and higher CHE/GDP tend to have longer survival rates.

## Figures and Tables

**Table 1:** Summary of the number, crude rank, ASR, and MIR of lip and oral cancer by region.

	New Cases			Deaths			MIR
Region	Number	CR	ASR	Number	CR	ASR	
Africa	13,324	1.0	1.7	9066	0.7	1.2	0.71
Asia	220,810	4.9	4.2	124,900	2.8	2.4	0.57
Europe	57,737	8.0	4.3	21,834	3.0	1.6	0.38
Latin America and the Caribbean	18,525	2.9	2.6	7050	1.1	1.0	0.38
North America	25,354	7.1	4.2	4424	1.2	0.7	0.17
Oceania	4163	10.2	7.5	895	2.2	1.5	0.22

**Table 2:** Summary of HDI, CHE, cancer incidence, cancer mortality, and MIR in lip and oral cancer (N = 61).

Country	HDI	CHE		Incidence			Mortality			MIR
		Per Capita	% of GDP	Number	CR	ASR	Number	CR	ASR	
Argentina	0.830	998	6.8	1357	3.1	2.5	512	1.2	0.9	0.39
Australia	0.938	4934	9.4	2682	11.1	6.9	378	1.6	0.9	0.14
Austria	0.914	4536	10.3	488	5.7	3.0	232	2.7	1.4	0.47
Bahrain	0.838	1190	5.2	16	1.0	1.5	10	0.6	1.0	0.64
Belarus	0.817	352	6.1	653	7.0	4.1	316	3.4	2.0	0.49
Belgium	0.919	4228	10.5	923	8.3	4.7	305	2.7	1.4	0.33
Brazil	0.761	780	8.9	9902	4.7	4.0	3965	1.9	1.6	0.40
Bulgaria	0.816	572	8.2	459	6.7	3.4	164	2.4	1.2	0.36
Canada	0.922	4508	10.4	2633	7.3	3.9	594	1.6	0.8	0.22
Chile	0.847	1102	8.1	275	1.5	1.1	113	0.6	0.4	0.42
Colombia	0.761	374	6.2	775	1.6	1.4	308	0.6	0.5	0.39
Costa Rica	0.794	929	8.1	78	1.6	1.2	35	0.7	0.5	0.44
Croatia	0.837	852	7.4	291	7.2	3.8	117	2.9	1.5	0.40
Cuba	0.778	826	10.9	1238	11.0	6.2	380	3.4	1.8	0.31
Cyprus	0.873	1563	6.8	29	2.5	1.6	8	0.7	0.5	0.27
Czechia	0.891	1284	7.3	859	8.3	4.3	361	3.5	1.8	0.42
Denmark	0.930	5497	10.3	397	7.1	3.8	125	2.2	1.1	0.31
Ecuador	0.758	530	8.5	217	1.3	1.2	82	0.5	0.5	0.38
Egypt	0.700	157	4.2	1295	1.3	1.6	352	0.4	0.4	0.28
Estonia	0.882	1112	6.5	79	6.2	3.3	40	3.1	1.7	0.50
Fiji	0.724	175	3.6	27	3.0	2.9	11	1.2	1.2	0.40
Finland	0.925	4005	9.4	348	6.5	3.0	116	2.2	0.9	0.34
France	0.891	4026	11.1	6815	10.8	6.2	1516	2.4	1.3	0.22
Germany	0.939	4592	11.2	7271	9.1	4.4	2311	2.9	1.3	0.32
Iceland	0.938	4375	8.6	13	3.9	2.3	3	0.9	0.5	0.23
Ireland	0.942	4757	7.8	275	5.8	3.7	74	1.6	0.9	0.28
Israel	0.906	2756	7.4	155	1.9	1.4	51	0.6	0.4	0.32
Italy	0.883	2700	9.0	3500	6.1	2.7	1184	2.1	0.9	0.34
Jamaica	0.726	294	5.9	52	1.8	1.5	15	0.5	0.4	0.29
Japan	0.915	3733	10.9	8138	6.7	2.8	2496	2.1	0.7	0.31
Kuwait	0.808	1169	4.0	59	1.4	2.0	33	0.8	1.1	0.56
Latvia	0.854	784	5.8	239	12.7	6.8	113	6.0	3.2	0.47
Lithuania	0.869	923	6.5	188	6.7	3.7	114	4.1	2.3	0.61
Luxembourg	0.909	6236	6.0	45	7.8	4.8	10	1.7	1.0	0.22
Malaysia	0.804	386	4.0	640	2.0	2.0	302	1.0	1.0	0.48
Malta	0.885	2304	9.6	18	4.2	1.9	6	1.4	0.4	0.33
Mauritius	0.796	506	5.5	62	4.9	3.3	28	2.2	1.5	0.45
Netherlands	0.934	4746	10.7	1418	8.5	4.4	257	1.5	0.7	0.18
New Zealand	0.921	3554	9.3	264	5.7	3.4	58	1.2	0.7	0.21
Norway	0.954	7464	10.0	362	6.9	3.7	68	1.3	0.7	0.19
Oman	0.834	636	3.8	65	1.3	2.0	31	0.6	1.1	0.49
Philippines	0.712	127	4.4	1372	1.3	1.6	673	0.6	0.8	0.48
Poland	0.872	797	6.3	3203	8.6	4.8	1505	4.0	2.2	0.47
Portugal	0.850	1722	9.0	817	8.2	4.3	269	2.7	1.4	0.33
Qatar	0.848	2030	3.1	26	1.0	2.0	14	0.5	1.4	0.54
Russian Federation	0.824	524	5.6	9340	6.6	4.0	5443	3.8	2.3	0.58
Serbia	0.799	491	9.4	701	8.1	4.9	261	3.0	1.7	0.37
Singapore	0.935	2280	4.3	199	3.5	1.9	85	1.5	0.8	0.43
Slovakia	0.857	1108	6.9	541	10.1	6.0	224	4.2	2.5	0.42
Slovenia	0.902	1772	8.5	135	6.7	3.5	47	2.3	1.1	0.34
South Africa	0.705	471	8.2	1328	2.3	2.7	829	1.4	1.7	0.61
South Korea	0.906	2013	7.4	1467	2.9	1.6	507	1.0	0.5	0.34
Spain	0.893	2354	9.2	3843	8.6	4.0	969	2.2	1.0	0.26
Sweden	0.937	5600	11.0	556	5.7	2.8	145	1.5	0.7	0.26
Switzerland	0.946	9818	12.1	639	7.7	4.0	184	2.2	1.1	0.29
Thailand	0.765	217	3.8	4169	6.1	3.8	2159	3.2	2.0	0.52
Trinidad and Tobago	0.799	1146	6.0	42	3.1	2.2	20	1.5	1.1	0.48
Ukraine	0.750	125	6.1	3358	7.8	4.4	2058	4.7	2.8	0.60
United Kingdom	0.920	4356	9.9	5645	8.7	4.9	1443	2.2	1.1	0.25
United States of America	0.920	9536	16.8	22715	7.1	4.2	3830	1.2	0.7	0.17
Uruguay	0.808	1281	9.2	149	4.4	2.9	70	2.1	1.3	0.48

## Data Availability

The datasets used and/or analyzed during the current study are publicly available in the Global Cancer Observatory (GLOBOCAN) database (https://gco.iarc.fr/today/, accessed date: 11 October 2020), United Nations Development Program/Human Development Report Office (http://hdr.undp.org/en, accessed date: 11 October 2020) and World Health Statistics database (https://www.who.int/gho/publications/world_health_statistics/en/, accessed date: 11 October 2020).
